# Adherence to antihypertensive medication and its associated factors among patients with hypertension attending a tertiary hospital in Kathmandu, Nepal

**DOI:** 10.1371/journal.pone.0305941

**Published:** 2024-07-03

**Authors:** Sunita Sharma, Chitra Raj Sharma, Suraj Sharma, Sajiva Aryal, Buna Bhandari

**Affiliations:** 1 Central Department of Public Health, Institute of Medicine, Tribhuvan University, Kathmandu, Nepal; 2 National Academy of Medical Sciences, Bir Hospital, Kathmandu, Nepal; 3 Kathmandu Medical College, Kathmandu, Nepal; 4 Department of Global Health and Population, Harvard T.H Chan School of Public Health, Boston, Massachusetts, United States of America; Patan Academy of Health Sciences, NEPAL

## Abstract

Hypertension is a major risk factor for cardiovascular disease, which is the leading cause of premature mortality and morbidity globally. Despite the evidences of the availability of effective treatment for hypertension, its management remains suboptimal. Medication adherence is the most crucial factor for blood pressure control. It is important to identify the factors associated with adherence to antihypertensive treatment for better management. Hence, this study assessed the level of antihypertensive medication adherence and its associated factors among patients with hypertension visiting a tertiary-level hospital in Kathmandu, Nepal. An analytical cross-sectional study was carried out among 308 diagnosed patients with hypertension who were prescribed antihypertensive medication. The Morisky Medication Adherence Scale (MMAS-8) was used to assess medication adherence. Data was collected through face-to-face interviews and analysed using SPSS v26. A bivariate and multivariate logistic regression model was used to assess the factors associated with low medication adherence. More than half (61%) of the study participants had moderate to high levels of medication adherence. Upon bivariate analysis, there was a significant association between presence of side effects, blood pressure status, forgetfulness, high cost, fear of taking medicine lifelong and irregular follow-up with a low level of adherence. Upon multivariate the logistic regression analysis, forgetfulness [Adjusted Odd’s Ratio (AOR) 22.5, 95% Confidence Interval(CI) 10.56–47.86], high cost (AOR 3.8, 95%CI 1.25–11.60) and fear of taking medicines lifelong (AOR 6.04, 95%CI 2.96–12.33) were found to be associated factors of low level of adherence. There is an urgency to develop evidence-based strategies to improve the level of adherence to antihypertensive medications among patients with hypertension. Strategies like reminder messaging, setting alarms, expanding the scope of national health insurance and proper counselling to reduce fear could help to improve medication adherence. Hence, the feasibility and effectiveness of such intervention should be explored in future studies.

## Introduction

Globally, hypertension (HTN) is the leading risk factor for death and disability, contributing to 10 million deaths annually in 2019. More than 20% of adults worldwide suffer from hypertension, and 75% of them reside in Low-middle-income countries (LMICs) [1, 2]. Hypertension is a significant contributing factor in about 47% of deaths from heart disease and 54% of fatalities from stroke [3]. In addition, Nepal’s rising hypertension prevalence is another concern. Surveys carried out in different parts of Nepal between 2011 and 2016 indicated escalating prevalence of hypertension over the decades, which rose by 6% in Nepal between 2000 and 2020, according to a systematic review by Dhungana et al. in 2021 [4].

Hypertension is inadequately controlled even after its diagnosis despite the availability of effective treatment in Nepal [5]. Several studies have reported low medication adherence, irregular follow-up, a lack of uniform treatment protocols, and healthcare practitioners’ inability to convey messages as barriers to hypertension management in Nepal [4, 5]. According to World Health Organisation (WHO), at least 50% of diagnosed patients with Hypertension (HTN) do not take antihypertensive medication as prescribed [6]. Adherence to prescribed pharmacotherapies is an important predictor of optimal blood pressure (BP) control and subsequent reduction in the complication burden due to hypertension [7].

Poor medication adherence results in significant disease worsening, an increase in avoidable hospital admission rates, and extended hospital stays, which decrease treatment efficacy and increase the risk of cardiovascular events. According to several studies in clinical setting, non-adherence to anti-hypertensive treatment has also hampered drug efficiency [8–10].

Recent review provided evidence of a huge gap in the cascade of hypertension care in Nepal [11]. The study done in the community setting of Nepal reported that 37.5% [12] of hypertensive patients had a high level of adherence whereas in a hospital setting, only 23.9% [13] of hypertensive patients had a high level of adherence. The recent meta-analysis of Nepal reported that half of the hypertensive population of Nepal is non-adherent to their anti-hypertensive medications, posing a higher risk of cardiovascular events [14].

Therefore, it is crucial to identify the factors associated with adherence to antihypertensive medication to improve the control of blood pressure among patients with hypertension in Nepal [14]. Several studies have reported the presence of co-morbidity, alcohol consumption, self-purchasing of medications [7, 14], forgetfulness [13, 14], unavailability of hypertensive medicines at the nearest health facilities [15], large number of medications prescribed, irregular follow-up [16, 17], etc., were all linked to non-adherence to antihypertensive medication and all identified factors were modifiable. Although several studies have shown the prevalence of hypertension, level of medication adherence and factors associated with adherence to antihypertensive medications in some parts of Nepal [13, 15–17], there is limited evidence on medication adherence at the tertiary level hospital in Nepal.

Therefore, this study was conducted to assess the level of medication adherence and its associated factors among patients with hypertension attending Manmohan Cardiothoracic Vascular and Transplant Centre, a cardiac tertiary center hospital in Nepal, to inform the future contextual intervention design and implementation.

## Materials and methods

### Study design and setting

This was a hospital-based analytical cross-sectional study conducted at Manmohan Cardiothoracic Vascular and Transplant Center (MCVTC), a tertiary care hospital of Kathmandu district under the Bagmati Province of Nepal. This hospital is located in the capital city of Nepal within the premises of Tribhuvan University and Teaching Hospital, a leading healthcare institution in the country, for more than two decades. On average, 80–120 patients attend the outpatient department (OPD) each day for Hypertension and various other heart conditions based on hospital records.

#### Study population

This study included the diagnosed patients with hypertension who visited the cardiology Out Patient Department (OPD) of Manmohan Cardiothoracic Vascular and Transplant Center (MCVTC) in Nepal.

All patients diagnosed with hypertension and prescribed with at least one antihypertensive medication at least 6 months before and aged 18 years and above were eligible to be included in the study. Pregnant, post-partum women and the patients who had severe physical and mental problems limiting the participation in the study were excluded.

#### Sample size

The total calculated sample size 308 was determined using the statistical formula by Cochran. The prevalence (p) of the study is adapted from a recent hospital-based cross-sectional study by Khadka et al., 2021, that reported a 23.9% prevalence of medication adherence among hypertensive patients [13] with a 95% confidence interval and a 5% absolute margin of error.

#### Sampling technique

Systematic random sampling was used to select the study participants. Study participants were selected from the OPD of MCVTC in the month of December 2022. An approximate number of diagnosed hypertensive patients under medication visiting cardiology OPD of MCVTC within one month (December) was identified from an average of previous OPD records of 3 years. N = 948+898+968/3 = 938/month. The number obtained (N) was divided by the calculated sample size (n) to get the kth interval, and systematic random sampling was done. k = 938/308 = 3. Taking 25 working days, the approximate per day flow of patients in OPD = 938/25 = 37. The first sample was selected by simple random sampling (lottery method). Other samples participated in every 3^rd^ interval by circular systematic random sampling method until the required sample size was achieved. If the selected sample did not fulfill the inclusion criteria of the study, then, consecutive patient fulfilling inclusion criteria was selected as a sample for the data collection.

#### Data collection tools and technique

The data was collected by a structured interview schedule by the interviewer with the help of two trained enumerators.

The schedule was divided into five sections: a) Information related to Socio-demographic Characteristics, b) Information related to clinical characteristics of patients, c)Information related to behavioural characteristics of patients, d) Information related to Adherence to antihypertensive medication by using 8-item Morisky Medication Adherence Scale (MMAS-8), e) Screening questions for standard blood pressure measurement as per the guideline provided by CDC 2021 and Measurement of blood pressure using The Omron HBP-1300 upper arm automatic blood pressure monitor.

Information related to Adherence to antihypertensive medication by using the 8-item Morisky Medication Adherence Scale (MMAS-8), which is a widely used 8-item validated tool, was used to measure antihypertensive medication adherence. The MMAS-8 diagnostic adherence assessment instrument has a range of 0 to 8. Low adherence is 0 to <6, moderate adherence is from 6 to <8, and high adherence is 8 [18–20]. Permission to use standard tool (MMAS-8) was taken from Dr. Arnold Morisky and the team [Certificate Number: 6064-8836-6630-2307-1825]. Pre-testing the tool in 10% of the sample size in hypertensive patients under medications was done among patients attending the medical Out Patient Department (OPD) of Tribhuvan University Teaching Hospital (TUTH).

#### Independent variables

Socio-demographic characteristics: Age, Sex, Ethnicity, Education, Employment status, Marital status.

Clinical characteristics: Blood Pressure status, Duration of diagnosis Family history, Co-morbidity, Number of medications prescribed, frequency of medication use, presence of side-effects.

Behavioral characteristics: Use of alternative medicine, forgetfulness, missed medication due to high cost, regular follow-up, Fear of taking medications lifelong.

#### Blood pressure measurement

Blood pressure (BP) was measured following the standard blood pressure measurement procedure as per the Centers for Disease Control and Prevention (CDC) guideline [21]. Following the CDC guideline, participants were asked about their last meal intake and data was collected after an hour of their last meal intake. Additionally, they were asked about their smoking and alcohol habits. All the participants in the study had not consumed alcohol or smoked before 30 minutes at the time of data collection.

Before the BP measurement, participants were instructed to rest in a sitting position for at least 10 minutes. BP was measured using a regularly calibrated random-zero sphygmomanometer in the right arm. For all study participants, BP measurements were conducted 2 times in a sitting posture, and the mean of two BP readings was calculated to determine whether BP targets were achieved.

Controlled/Targeted Blood Pressure: Blood pressure was considered controlled when the average of two readings in the hospital setting was below 140/90 mm Hg for all hypertensive patients, as per the International Society of Hypertension (ISH) 2020 guideline [22].

Uncontrolled or High Blood Pressure: An average of two readings ≥140/90 mmHg taken one minute apart suggested uncontrolled hypertension as per ISH guideline 2020 [22]. Data collection began on December 1^st^ 2022 and was completed on December 31st, 2022. The first author (SS) was involved in the data collection and supervised two trained enumerators.

#### Data management and analysis

The collected data was assessed daily for completeness and consistency of information. Then, the data was systematically coded and entered into IBM SPSS version 26, where cleaning and editing were done. The frequency distribution of each variable was examined to check for errors.

Data analysis was done in three stages. In the first stage, descriptive analyses (frequency and percentage) were used to report the socio-demographic characteristics, clinical characteristics and behavioural characteristics of the study participants. Frequency tables were generated for categorical variables, while mean and standard deviation were calculated for continuous variables.

The second stage of the analysis involved testing for the association through bivariate analysis of socio-demographic, clinical, and behavioural variables with the level of adherence, and p-values at a 95% level of confidence was reported.

The third stage involved the comparison of the independent variable with the dependent variable (low level of adherence) using logistic regression.

#### Validity

The translation validity of the tool was ascertained by translating the tool into Nepali language and forward and backward translation of the tool was done by two Nepali teachers from National Institute of Science and Technology (NIST) by strictly following the translation guidelines from World Health Organisation (WHO) [23], which was later reviewed by the supervisors. Pretesting was done in 10% of the sample size in hypertensive patients under medications attending the Medical Out Patient Department (OPD) of Tribhuvan University Teaching Hospital (TUTH). After pretesting, some confusing words in Nepali tool were re-written and ambiguous words with unclear meanings were omitted.

#### Reliability

The reliability of the tool was established by pre-testing the tool in conveniently selected 32 samples of attending medical OPD of TUTH. First, the participants were interviewed by the researcher, and on the subsequent day, the same participant was interviewed by the enumerator. The responses were compared to check the consistency of the responses received. During pretesting, the translation and understanding of the questionnaire were checked, and corrections were made to the wording of questions and overall layout to clarify meaning. Internal consistency of adherence items was ascertained by calculating Cronbach’s alpha using IBM SPSS. The Cronbach’s alpha value of 0.72 was obtained. The tool was translated into the Nepali language for administration and back-translated into English. To get consistent and reliable information, enumerators were provided with a one-day orientation about the purpose of the study, structure of the questionnaire, method and process of data collection, ethical considerations and communication skills. The enumerators were closely supervised throughout the process of data collection.

#### Ethical considerations

Ethical approval was obtained from the Institutional Review Committee (IRC) of the Institute of Medicine [Ref: 264(6–11) E2 2079/080]. Formal permission was also obtained from the administration section of MCVTC for conducting the study. The permission for the use of the standard tool (MMAS-8) was taken from the original developer of the tool, Dr. Arnold Morisky, and the team [Certificate Number: 6064-8836-6630-2307-1825].

Written informed consent, after an explanation about the study, was obtained from the study participants. Privacy during the data collection was maintained by collecting data in a separate room from the Out Patient Department (OPD).

## Results

A total of 320 patients were approached for data collection; 12 patients were excluded as they did not fulfill the inclusion criteria, after excluding 12 patients, and 308 patients were included in the study. The mean age of participants was 58.1 years with standard deviation of 11.7 years. The age range of participants was between 23 to 87 years, with nearly half of them (47.4%) in the age group 60 years and above, followed by 31.5% from the age group 50–59 years, with around half (51.3%) females. More than half of the participants (52.3%) in this study were Brahmin/Chhetri, and 58.1% were literate, and the majority of them (72.0%) were self-employed, and the majority (87.3%) of the study participants were married as presented in Table 1.

**Table 1:** Socio-demographic characteristics of study participants (n = 308).

Characteristics	Number	Percentage
**Completed age**		
>50 years	65	21.1
50–59 years	97	31.5
≥60 years and above	146	47.4
Mean age ± SD	58.1±11.7	
**Sex**		
Female	158	51.3
Male	150	48.7
**Ethnicity**		
Brahmin/Chhetri	161	52.3
Janajati	115	37.3
Others*	32	10.4
**Educational status**		
Literate	179	58.1
Illiterate	129	41.9
**Employment status**		
Self employed	222	72.0
Private	50	16.3
Government	36	11.7
**Marital status**		
Married	269	87.3
Unmarried	39	12.7

Others* in Ethnicity includes Dalit, Madhesi and Muslim

As presented in Table 2, more than half (61%) of the participants had moderate to high levels of adherence, and less than two-fifths (39%) of the study participants had low levels of adherence.

**Table 2:** Level of adherence of the study participants (n = 308).

Level	Number	Percentage	95%CI
Moderate to high level of adherence (MMAS score 6–8)	188	61.0	(0.55–0.66)
Low level of adherence (MMAS score <6)	120	39.0	(0.33–0.44)

The MMAS-8© Scale, content, name, and trademarks are protected by US copyright and trademark laws. Permission to use the scale and its coding is required. A license agreement is available from MMAR, LLC., www.moriskyscale.com.

There was no significant association of socio-demographic variables age, sex, ethnicity, education, employment status and marital status of patients with level of adherence as reported in Table 3.

**Table 3:** Association of socio-demographic characteristics of participants with level of adherence (n = 308).

Characteristics	Categories	Adherence level		P-value
		Moderate-High	Low	
Age group	> 50	43(66.2%)	22(33.8%)	0.559
	50–59	56(57.7%)	41(42.3%)	
	≥60	89(61%)	57(39%)	
Sex	Female	99(62.7%)	59(37.3%)	0.550
	Male	89(61.0%)	61(39.0%)	
Ethnicity	Brahmin/Chhetri	106 (65.8%)	55 (34.2%)	0.146
	Janajati	66 (57.4%)	49 (47.6%)	
	Others*	16 (50%)	16(50%)	
Education	Literate	116 (64.8%)	63 (35.2%)	0.110
	Illiterate	72 (55.8%)	57 (44.2%)	
Employment status	Self-employed	134(60.4%)	88(39.6%)	0.893
	Private	32(64%)	18(36%)	
	Government	22(61.1%)	14(38.9%)	
Marital status	Married	168(62.5%)	101(37.5%)	0.181
	Unmarried	20(51.3%)	19(48.7%)	

Others* in Ethnicity include Dalit, Madhesi and Muslim

However, there was a significant association between the presence of side effects, blood pressure status, forgetfulness, missed medicines due to high cost, regular follow up and fear of taking medicines lifelong with a level of adherence as presented in Table 4.

**Table 4:** Association of clinical and behavioral characteristics of participants with level of adherence (n = 308).

Characteristics	Categories	Adherence level		P-value
		Moderate-High	Low	
Duration of diagnosis	Up to 5 years	71(60.7%)	46(39.3%)	0.920
	More than 5 years	117(61.3%)	74(38.7%)	
Family history	Yes	130(61%)	83(39%)	0.997
	No	58(61.1%)	37(38.9%)	
Co-morbidity	Yes	125(59.5%)	85(40.5%)	0.425
	No	63(64.3%)	35(35.7%)	
Number of Medications Prescribed	Only one	137(63.4%)	79(36.6%)	0.188
	More than one	51(55.4%)	41(44.6%)	
Frequency of use of medications	One time/day	131(63.9%)	74(36.1%)	0.146
	More than one time/day	57(55.3%)	46(44.7%)	
**Presence of any side-effects**	Yes	21(46.7%)	24(53.3%)	**<0.05** *
	No	167(63.4%)	96(36.5%)	
**Blood Pressure Status**	Controlled	111(66.9%)	55(33.2%)	**<0.05** *
	Uncontrolled	77(54.2%)	65(45.8%)	
Use of alternative medicine	Yes	18(46.2%)	21(53.8%)	0.41
	No	170(63.2%)	99(36.8%)	
**Forgetfulness**	Yes	53(33.5%)	105(66.5%)	**<0.001** *
	No	135(90%)	15(10%)	
**Missed medicines due to high cost**	Yes	6(16.2%)	31(83.8%)	**<0.001** *
	No	182(67.2%)	89(32.8%)	
**Regular follow-up**	Yes	155(67.1%)	76(32.9%)	**<0.001** *
	No	33(42.9%)	44(57.1%)	
**Fear of taking medications lifelong**	Yes	52(40.6%)	76(59.4%)	**<0.001** *
	No	136(75.6%)	44(24.4%)	

* Statistically significant at 95% level of confidence

Similarly, among the total respondents having side-effects, more than half (53.3%) had low level of adherence compared to 36.5% of respondents with absence of side-effects of medications. Among the respondents with uncontrolled blood pressure, nearly half of the participants (45.8%) had low level of adherence while only 33.2% of participants with controlled blood pressure had low adherence level.

In addition, more than half (66.5%) who forgot to take medicines had low adherence levels compared to only 10% of respondents with no forgetfulness.

Similarly, the majority of the participants (83.8%) who missed medicines due to high cost had low levels of adherence compared to only 32.8% of participants who did not miss medicines due to high cost.

More than half of the participants (57.1%) who had an irregular follow-up had a low level of adherence, while only 32.9% of participants who did regular follow-up had a low adherence level. More than half of the participants (59.4%) who feared taking medications lifelong had a low level of adherence compared to only 24.4% of participants who had no fear, as presented in Table 4.

Further, those six variables found significant at 5% significance level in bivariate analyses were entered into multivariate analysis. Multi-collinearity was assessed before performing logistic regression by utilizing Variance Inflation Factor (VIF). The decisive criterion for excluding variables from the regression model was set out as those with VIF values greater than 10. Variance inflation factor (VIF) was checked using collinearity diagnostics. No collinearity problems among the explanatory variables were identified (the highest observed VIF was 1.168). The goodness of fit of the regression model was tested by the application of the Hosmer and Lemeshow Chi-square test. Hosmer Lemeshow Chi-square statistic in the model showed no significant difference between the observed and predicted probabilities (p-value 0.607). This indicated a good model fit. Also, in the model, a Nagelkerke (pseudo) R Square value of 0.566 was observed, which indicates that 56.6 per cent of the variability in the adherence level was explained by the explanatory variables. The significance level to determine the statistical significance of independent variables is P-value <0.05.

The multivariate logistic regression analysis reported that forgetfulness, missed medications due to high cost, and fear of taking medications lifelong were significantly associated with low adherence to antihypertensive medication, as presented in Table 5.

**Table 5:** Factors associated with the level of adherence (n = 308).

Variables	Unadjusted OR	95% CI	P-value	Adjusted OR	95% CI	P-value
**Presence of side-effects**						
Yes	2.039	(1.04–3.980)	**<0.05** *	1.48	0.62–3.56	0.379
No	Ref.			Ref.		
**Forgetfulness**						
Yes	18.25	(9.22–36.12)	**<0.001** *	22.48	10.56–47.86	**<0.001** *
No	Ref.			Ref.		
**Missed medicines due to cost**						
Yes	11.53	(4.56–29.12)	**<0.001** *	3.80	1.25–11.60	**<0.01** *
No	Ref.			Ref.		
**Regular follow up**						
Yes	0.367	(.210-.640)	**<0.001** *	0.69	0.347–1.41	0.318
No	Ref.			Ref		
**Fear of taking medications lifelong**						
Yes	4.5	(2.67–7.5)	**<0.001** *	6.04	2.96–12.33	**<0.001** *
No	Ref.			Ref		
**Blood Pressure status**						
Controlled	0.565	(0.34–0.92)	**<0.05** *	1.57	0.83–2.98	0.165
Uncontrolled	Ref.			Ref		

* Statistically significant at 95% level of confidence

The participants who forgot to take antihypertensive medications were 22.5 times more likely to have a low level of adherence (AOR 22.5, 95%CI 10.56–47.86) compared to those who did not forget to take medications. Likewise, the participants who missed medicines due to high cost were 3.8 times more likely to have a low level of adherence (AOR 3.8, 95%CI 1.249–11.6) compared to those who did not miss medicines due to cost. The participants who feared taking antihypertensive medication lifelong were six times more likely to have low adherence (AOR 6.04, 95%CI 2.960–12.33) than those without fear as presented in Table 5.

## Discussion

Adherence to antihypertensive medication is an important factor in controlling blood pressure among diagnosed patients with hypertension. A multinational cross-sectional study reported a significant gap in the awareness level and control of blood pressure among patients with hypertension [24].

This study revealed that 61% of patients with hypertension had moderate to high levels of adherence to antihypertensive medications. This study identified associated factors of low adherence as forgetfulness, missed medicines due to high cost, and fear of taking medicines lifelong.

The prevalence of moderate to high adherence in our study was 61%, at 95%CI (0.55–0.66). However, the community-based study [17] from the Eastern part of Nepal reported that among 154 hypertensive patients, 56.5% of patients were adherent to antihypertensive medications. This could be due to the measurement of adherence based on different criteria, along with variation in the subset of the population that served as the study sample. In addition, the previous study was community based, whereas ours is a hospital-based study. The results of our study is higher than what has been reported from Malaysia (53.4%) [25], Taiwan (47.5%) [26] and Bangladesh (41%) [27]. The adherence rate in this study is also higher than the study from the western part of Nepal, Pokhara (35.4%) [28]. However, more people of LMICs in Pakistan [29], 77%, were adherent to their medication than in this study. This may be due to differences in measurement criteria of adherence and difference in socio-demographic characteristics. MMAS 4 tool was used to assess the level of adherence in most of the studies, whereas we used MMAS-8 in our study. The MMAS-4 is a generic self-reported medication-taking behavior scale which consists of four items with a scoring scheme of “Yes” = 0 and “No” = 1. The items are summed to give a range of scores from 0 to 4 [30]. However, this study used the MMAS-8 which consists of 8 items containing question about medication adherence. Low adherence is 0 to <6, moderate adherence is from 6 to <8 and high adherence is 8. Nevertheless, it is important to have an optimum level of adherence for blood pressure control, so the findings of our study are crucial to address the issue of non-adherence.

The study found forgetfulness as one of the determinants of low levels of adherence to antihypertensive medications. The participants who forgot to take antihypertensive medications were 22.5 times more likely to have a low level of adherence compared to those who did not forget to take medicines. In a study conducted in a multispecialty tertiary care teaching hospital in India [31], it was reported that forgetfulness, the presence of side effects, and not taking medication timely are the reasons for low adherence. Forgetfulness was the top reported cause (72%) of unintended non-adherence, similar to our findings. It is also consistent with the findings from a hospital-based study done in five centres in Nepal [13] that reported 56% of the participants were non-adherent to antihypertensive therapy due to forgetfulness. These demand strategies to address forgetfulness, such as text or voice messages, and personalized reminders that can remind patients to improve their adherence to medications [32, 36].

This study found that those who missed medications due to high cost were 3.8 times more likely to have a low adherence than those who did not miss medicines due to cost. The findings of the study are similar to the findings from the National health survey from a nationally representative sample of US citizens [33], which reported that antihypertensive medication adherence and hypertension control are significantly associated with the inability to obtain antihypertensive medication due to financial constraints. The findings of the study are also consistent with the study done in eastern Nepal [17], which identified missed medicines due to high cost as one of the predictors of low level of adherence. It demands the strategies to reduce the high out of pocket expenditure in health such as increasing the coverage of national health insurance [34].

Further, this study identified fear of taking e lifelong antihypertensive medicines as the associated factors of a low level of adherence. The participants who feared taking antihypertensive medication lifelong were 6 times more likely to have a low level of adherence than those without fear. The findings of the study are consistent with the study done by Shrestha et al. [35] in 2019, which reported fear of taking medication for a lifetime was the most common reason for non-adherence. These fears could be due to a lack of enough information to clarify their misconceptions [35]. Hence, those disease misconceptions can be overcome with adequate information from reliable sources to patients.

Different measures, like a reminder messaging alarm to reduce forgetfulness, counselling to reduce misconceptions about medications, and improving patient education, have been found to be effective improving adherence to antihypertensive medications among patients with hypertension [32, 34–36]. However, the effectiveness and feasibility of those interventions in addressing the issue of non-adherence in this context should be explored in future studies.

## Conclusion

Although the study found that more than half of the participants had moderate to high adherence levels, it remains concerning that two-fifths of the participants had low levels of adherence to antihypertensive medications. To improve the medication adherence level among patients with hypertension, the identified factors, such as forgetfulness, missed medicines due to high cost, and fear of taking medicines lifelong, should be addressed.

## Supporting information

S1 Table Distribution of characteristics by clinical characteristics. (DOCX)

S2 Table Distribution of participants by behavioral characteristics. (DOCX)

S3 Table Output table for collinearity diagnostics by VIF. (DOCX)
