# Supplementary material for: Adherence to antihypertensive medication and its associated factors among patients with hypertension attending a tertiary hospital in Kathmandu, Nepal
Source: PLoS One. 2024 Jul 3;19(7):e0305941. doi: 10.1371/journal.pone.0305941 (PMC11221664; doi:10.1371/journal.pone.0305941)
Supplement: S1 Table — (DOCX) [file pone.0305941.s001.docx]

**Distribution of participants by medical and clinical characteristics**

More than half (53.9%) of participants had blood pressure under control. More than half (62%) of study participants were diagnosed with hypertension for more than 5 years. Similarly, proportion of participants having family history of hypertension was 69.2% and co-morbidities were present among more than half (68.2%) of the respondents. Majority of participants (70.1%) had only one medication prescribed. More than half of the study participants (66.6%) took medication only one time a day (OD). Most of the study participants (85.4%) did not report any side effects of the antihypertensive medications they are taking as presented in S1 Table.

**S1 Table. Distribution of characteristics by clinical characteristics (n=308)**

| **Characteristics** | **Number** | **Percentage** |
| --- | --- | --- |
| **Blood Pressure status** | | |
| Controlled BP | 166 | 53.9 |
| Uncontrolled BP | 142 | 46.1 |
| **Duration of diagnosis** | | |
| Up to 5 years | 117 | 38.0 |
| More than 5 years | 191 | 62.0 |
| **Family history of hypertension** | | |
| Yes | 213 | 69.2 |
| No | 95 | 30.8 |
| **Presence of co-morbidity** | | |
| Yes | 210 | 68.2 |
| No | 98 | 31.8 |
| **Number of medicines prescribed** | | |
| Only one | 216 | 70.1 |
| More than one | 92 | 29.9 |
| **Frequency of medication use** | | |
| One time/day | 205 | 66.6 |
| More than one time/day | 103 | 33.4 |
| **Presence of side effects** | | |
| Yes | 45 | 14.6 |
| No | 263 | 85.4 |
