# Supplementary material for: Adherence to antihypertensive medication and its associated factors among patients with hypertension attending a tertiary hospital in Kathmandu, Nepal
Source: PLoS One. 2024 Jul 3;19(7):e0305941. doi: 10.1371/journal.pone.0305941 (PMC11221664; doi:10.1371/journal.pone.0305941)
Supplement: S2 Table — (DOCX) [file pone.0305941.s002.docx]

**Distribution of participants by behavioral characteristics**

Majority of the participants (87.3%) did not use any other alternative medications. More than half (51.3%) of the respondents reported forgetfulness in taking their medications. Less than one-eighth participants (12%) missed medicines due to high cost. About three-fourth (75%) participants came for regular follow up. Less than half (41.6%) of the study participants had fear of taking antihypertensive medications lifelong as reported in S2 Table.

**S2 Table. Distribution of participants by behavioral characteristics (n=308)**

| **Characteristics** | **Number** | **Percentage** |
| --- | --- | --- |
| **Use of alternative medicine** | | |
| Yes | 39 | 12.7 |
| No | 269 | 87.3 |
| **Forgetfulness** | | |
| Yes | 158 | 51.3 |
| No | 150 | 48.7 |
| **Missed medicines due to high cost** | | |
| Yes | 37 | 12.0 |
| No | 271 | 88.0 |
| **Regular follow up** | | |
| Yes | 231 | 75.0 |
| No | 77 | 25.0 |
| **Fear of taking medicine lifelong** | | |
| Yes | 128 | 41.6 |
| No | 180 | 58.4 |
