# Supplementary material for: Adherence to antihypertensive medication and its associated factors among patients with hypertension attending a tertiary hospital in Kathmandu, Nepal
Source: PLoS One. 2024 Jul 3;19(7):e0305941. doi: 10.1371/journal.pone.0305941 (PMC11221664; doi:10.1371/journal.pone.0305941)
Supplement: S3 Table — (DOCX) [file pone.0305941.s003.docx]

**S3 Table. Output table for collinearity diagnostics By VIF**

| **Coefficients** | | | | | | | |
| --- | --- | --- | --- | --- | --- | --- | --- |
| Model | | Standardized Coefficients | Sig. | 95.0% Confidence Interval for B | | Collinearity Statistics | |
|  |  | Beta |  | Lower Bound | Upper Bound | Tolerance | VIF |
| 1 | (Constant) |  | .000 | 2.413 | 3.333 |  |  |
|  | Presence of any side-effects | -.012 | .803 | -.148 | .115 | .966 | 1.035 |
|  | Ever Forgottten to take medicine | -.493 | .000 | -.576 | -.383 | .916 | 1.091 |
|  | Ever Missed medicines due to high cost | -.107 | .040 | -.340 | -.008 | .861 | 1.162 |
|  | Fear of taking antihypertensive medications lifelong | -.273 | .000 | -.370 | -.172 | .892 | 1.121 |
|  | Regular follow-up | .043 | .394 | -.064 | .163 | .932 | 1.072 |
|  | Information about regular medication use from Family Members | -.024 | .621 | -.126 | .075 | .949 | 1.054 |
| Dependent Variable: Level of adherence (moderate-high and low category) | | | | | | | |

**Output table for multivariate analysis**

**Block 1: Method = Enter**

| **Omnibus Tests of Model Coefficients** | | | | |
| --- | --- | --- | --- | --- |
|  | | Chi-square | Df | Sig. |
| Step 1 | Step | 166.205 | 6 | .000 |
|  | Block | 166.205 | 6 | .000 |
|  | Model | 166.205 | 6 | .000 |

| **Model Summary** | | | |
| --- | --- | --- | --- |
| Step | -2 Log likelihood | Cox & Snell R Square | Nagelkerke R Square |
| 1 | 245.636^a^ | .417 | .566 |
| a. Estimation terminated at iteration number 5 because parameter estimates changed by less than .001. | | | |

| **Hosmer and Lemeshow Test** | | | |
| --- | --- | --- | --- |
| Chi-square | df | Sig. |  |
| 5.431 | 7 | .607 |  |

| **Variables in the Equation** | | | | | | | | |
| --- | --- | --- | --- | --- | --- | --- | --- | --- |
|  | | B | S.E. | Sig. | Exp(B) | 95% C.I.for EXP(B) | |  |
|  |  |  |  |  |  | Lower | Upper |  |
| Step 1^a^ | Presence of any side-effects(1) | .394 | .448 | .379 | 1.483 | .617 | 3.565 |  |
|  | Ever Forgottten to take medicine(1) | 3.113 | .385 | .000 | 22.486 | 10.565 | 47.859 |  |
|  | Ever Missed medicines due to high cost(1) | 1.337 | .569 | .019 | 3.807 | 1.249 | 11.610 |  |
|  | Regular follow-up(1) | -.357 | .358 | .318 | .699 | .347 | 1.410 |  |
|  | Fear of taking antihypertensive medications lifelong(1) | 1.799 | .364 | .000 | 6.043 | 2.960 | 12.336 |  |
|  | Blood Pressure Status of the patients(1) | .453 | .326 | .165 | 1.574 | .830 | 2.984 |  |
|  | Constant | -2.827 | .689 | .000 | .024 |  |  |  |
| Variable(s) entered on step 1: Presence of any side-effects, Ever Forgotten to take medicine, Missed medicines due to high cost, Regular follow-up, Fear of taking antihypertensive medications lifelong, Blood Pressure Status of the patients. | | | | | | | | |
